# Supplementary material for: Prediction of Hemorrhagic Transformation after Ischemic Stroke Using Machine Learning
Source: J Pers Med. 2021 Aug 30;11(9):863. doi: 10.3390/jpm11090863 (PMC8470833; doi:10.3390/jpm11090863)
Supplement: Supplementary file 1 [file jpm-11-00863-s001.zip › jpm-1344842-supplementary.pdf]

Table S1. Grid search parameters in each machine learning classifier.

	BLR	SVM	XGB	ANN
C	[0.001, 0.01, 0.1, 1, 10, 100]	[0.001, 0.01, 0.1, 1, 10, 100]		
gamma		[0.001, 0.01, 0.1, 1, 10, 100]		
kernel		rbf, linear		
n_estimator			range(5,30)	
max_depth			range(6,10)	
learning rate			[0.4, 0.45, 0.5, 0.55, 0.6]	
colsample_bytree			[0.6, 0.7, 0.8, 0.9, 1.0]	
optimizer				adam
loss				binary_crossentropy
batch_size				50
Epoch				250

BLR, binary logistic regression; SVM, support vector machine; XGB, extreme gradient boosting; ANN, artificial neural network.

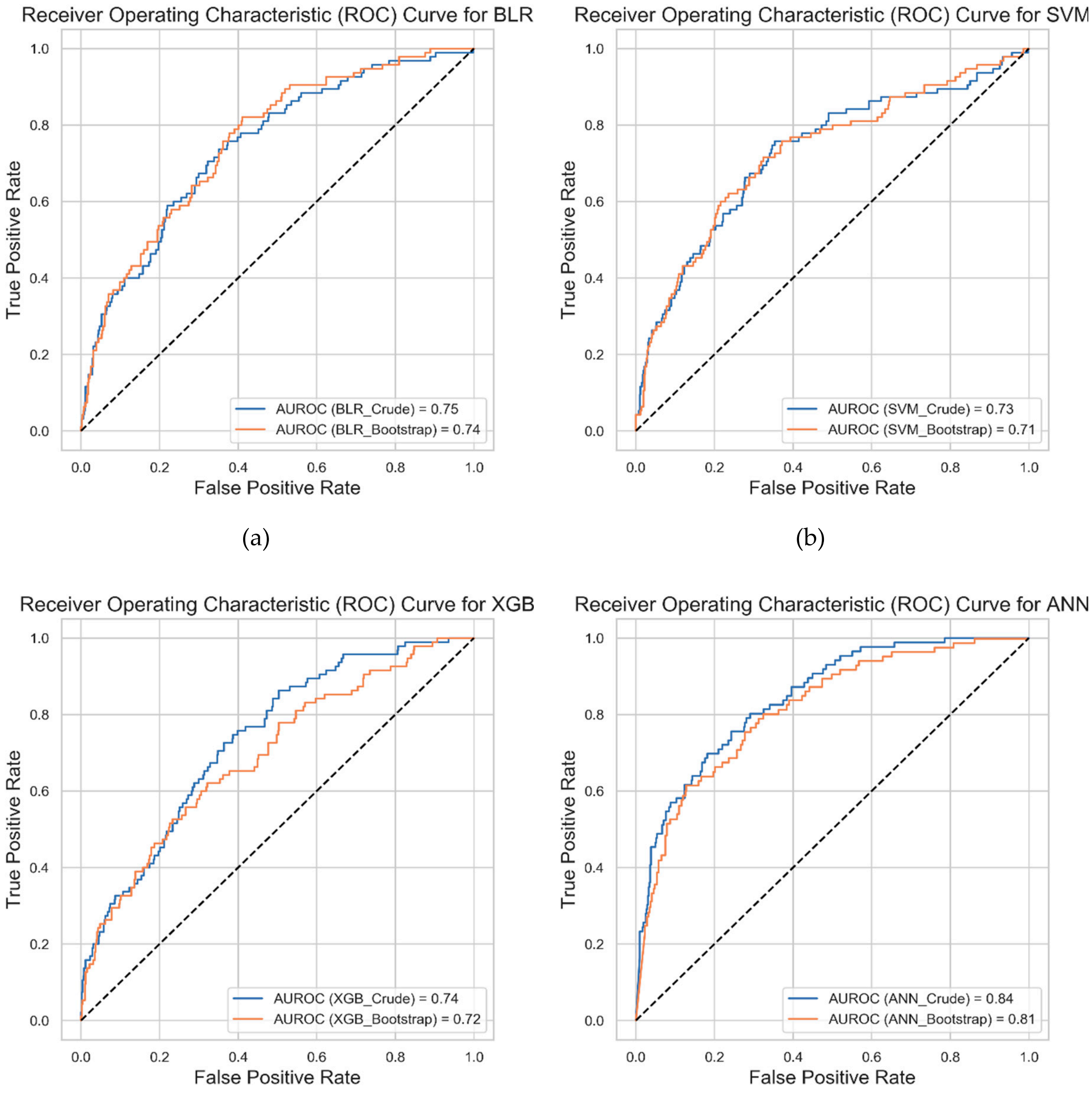

(a) (b)

(c) (d)

Figure S1. Result of the receiver operating characteristic curve of binary logistic regression (a) and support vector machine (b), extreme gradient boosting (c), and artificial neural network algorithm (d) before and after resampling strategy.

Each bootstrap resampled training set (120 different training set) was used to train each ML model. BLR, binary logistic regression; XGB, extreme gradient boosting; SVM, support vector machine; ANN, artificial neural network; AUROC, area under the receiver operating characteristic curve. 'Crude' refers to the case where the input variables were entered into the raw value, and the rest of them (normalizer, min-max, standard, and robust) were the different type of scaling methods for continuous variables.

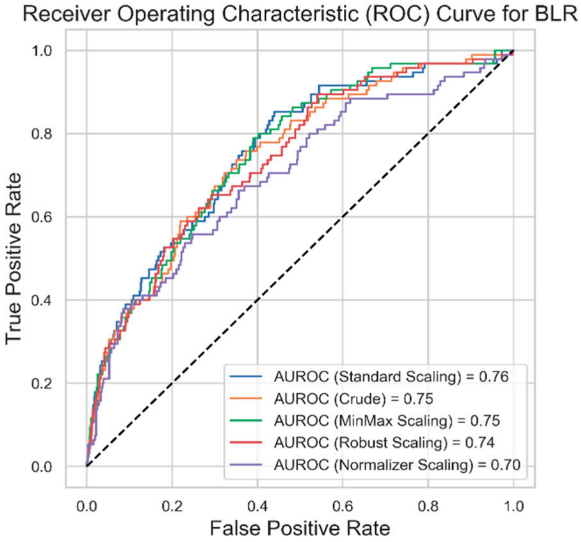

(a)

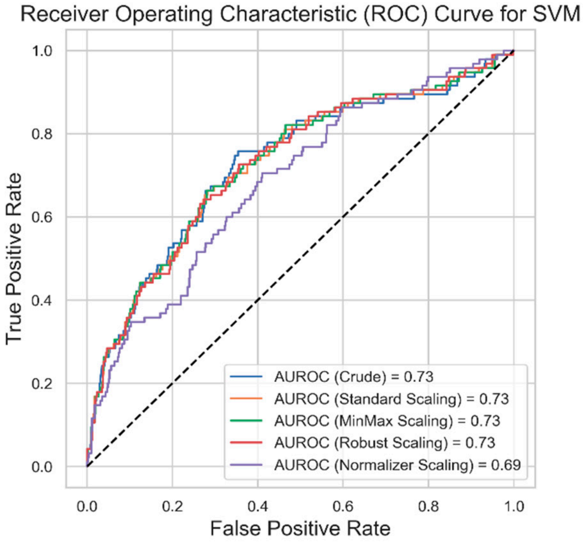

(b)

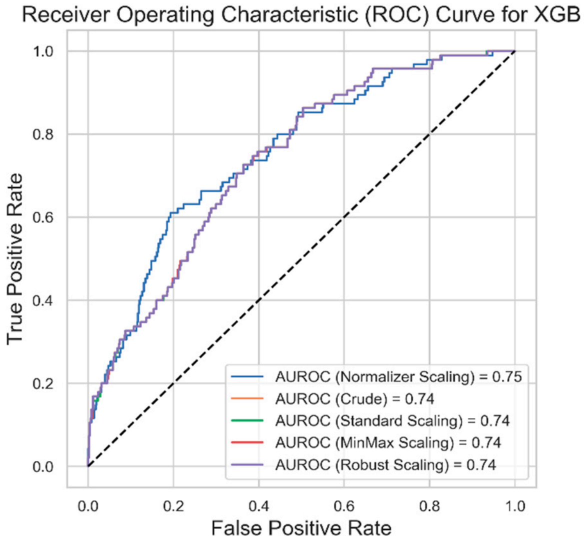

(c)

Figure S2. Result of the receiver operating characteristic curve of binary logistic regression (a), support vector machine (b), and extreme gradient boosting (c) before and after different scaling methods.

"Crude" refers to the case where the input variables were entered into the raw value, and the rest of the models were the results when continuous variables were applied to the model using various scaling methods (normalizer, min-max, standard, and robust).
